# Adolescents’ proxy reports on obesity-related parenting practices: factorial validity and reliability across four behavioral domains

**DOI:** 10.1186/s12889-022-12745-5

**Published:** 2022-02-16

**Authors:** Gabriel L. Fuligni, Christopher J. Gonzalez, Roger Figueroa

**Affiliations:** 1 College of Human Ecology, Division of Nutritional Sciences, Cornell University, 244 Garden Avenue, Ithaca, NY 14853 USA; 2 Division of General Internal Medicine, Weill Cornell Medicine, 338 E 66th Street, New York, NY USA; 3 Division of General Internal Medicine, 338 E 66th Street, New York, NY 10065 USA

**Keywords:** Obesity-related parenting, Parent-adolescent dyads, Confirmatory factor analysis, Actor-partner interdependence

## Abstract

**Background:**

Adolescents’ energy balance behaviors are precursors to obesity shaped by the practices or strategies that many parents implement. Although key stakeholders to their families, adolescents are rarely considered to report on these obesity-related parenting practices. The aim of this study is to assess the factorial and predictive validity of adolescents’ proxy-report of parents’ obesity-related parenting across four behavioral domains.

**Methods:**

This study used data from the Family Life, Activity, Sun, Health, and Eating (FLASHE) study. This study tests whether adolescents’ proxy reports about their parents’ obesity-related parenting are significantly associated with parents’ responses on their own obesity-related parenting, as well as whether these reports are significantly associated to parent-adolescent energy balance behaviors. Factorial validity was assessed using linear regression and Confirmatory Factor Analysis (CFA), whereas predictive validity was assessed using Actor-Partner Interdependence Modeling (APIM).

**Results:**

Regression results indicated that adolescents’ proxy report is significantly associated with parents’ report of their own parenting in all four domains (β = .59—.71; *p* < 0.05). CFA results indicated a final factor structure that loaded significantly onto hypothesized obesity-related parenting domains (β > .30) in both adolescents and parents. APIM results indicated that both parent- (β = .32; *p* < 0.05) and adolescent-(β = .21; *p* < 0.05) reported obesity parenting for fruit and vegetable consumption were associated with their own fruit and vegetable intake. In addition, adolescent-reported physical activity parenting was significantly associated with adolescent physical activity (β = 0.23; *p* < 0.05). Regarding partner effects, only parent-reported parenting for fruit and vegetable consumption were significantly associated with adolescent intake of fruit and vegetables (β = 0.15, *p* < 0.05) and adolescent-reported physical activity parenting was significantly associated with parental physical activity (β = 0.16, *p* < 0.05). Neither adolescent nor parent reported parenting were significantly associated with screen time or junk food intake outcomes. Each final obesity-related parenting scale had good internal consistency (*a* = .74-.85).

**Conclusions:**

We found that adolescent- and parent-reported obesity-related parenting were significantly associated, while adolescent-reported parenting were more explanatory of fruit and vegetable intake and physical activity than parent-reported parenting. These findings suggest that adolescent proxy reports may be a valid source of information on obesity-related parenting.

## Background

Over twenty percent of U.S. adolescents, defined here as 12- to 19-year-olds, are overweight or obese, and rates continue to increase nationwide [1]. Such alarming prevalence is consistent with global obesity trends among adolescents, driven by increases in energy-dense foods and increased engagement in sedentary behaviors [2]. Obesity puts adolescents at risk for a variety of adverse outcomes, including type 2 diabetes, metabolic syndrome, and cardiovascular disease [3, 4]. Adolescents with obesity are also more likely to experience social stigma and psychological comorbidities, including depression, emotional and behavioral disorders, and low self-esteem [5]. Obesity during adolescence greatly increases the risk of obesity in adulthood, due in part to impacting self-regulatory processes in this key period of brain development and leads to a greater risk of cardiovascular disease and several forms of cancer [3, 6, 7].

A variety of socio-ecological determinants can contribute to the development of obesity, including energy balance behaviors (i.e., diet and physical activity), psychosocial factors such as abuse, depression, and anxiety, and environmental factors, such as neighborhood walkability, fast food density, and access to healthy food options [2, 8–12]. Put simply, weight gain is caused by energy intake exceeding energy expenditure [13]. Given the critical impact of adolescent obesity on health and its growing prevalence globally and nationally, research on understanding additional ways to tackle this public health problem requires prioritization. This study focuses on the most proximal determinant of adolescent obesity, energy balance behaviors, and how they are shaped by the family context through parenting practices.

Being a primary agent of socialization, the family has great influence on the learning and development of behavior patterns among adolescents and is therefore an important area of study of adolescent obesity research [14]. Parents and their rearing practices have an important role in determining the energy balance behaviors of their adolescent children, both in terms of dietary intake and physical activity. Research has shown that parenting practices such as setting limitations on screen time, valuing and providing logistical support for being physically active, and parents themselves engaging in physical activity are associated with adolescents’ physical activity [15–19]. Similarly, adolescents’ diet is associated with parental encouragement, family eating rules, food availability at home, and parental diet [20, 21]. These findings highlight that parenting practices may have great influence on adolescents’ energy balance behaviors and indicate that investigating family influences on obesity is vital to understanding and preventing obesity among adolescents.

Obesity-related parenting practices are the focus of this study. Obesity-related parenting practices are the strategies that parents use to encourage their children’s dietary, physical activity, and screen-viewing behaviors. [22]. Although valid instruments for assessing obesity-related parenting practices exist, there are some notable gaps in the way parenting practices are currently measured [23]. In seven studies examining obesity-related parenting practices on adolescents’ energy balance behaviors, researchers focus on physical activity or diet as study outcomes, but not both within the same study [15, 24–29]. Also, the majority of these studies account for reports from either parents or adolescents only, with the exception of one study with a sample of 104 adolescent-parent dyads [24]. Systematic review evidence shows that out of 72 obesity-related parenting practices (i.e., food parenting) studies investigated, 11 included no validity evidence, most of the studies only demonstrated construct validity, only ten reported confirmatory factor analyses, and only two demonstrated criterion validity [30]. Noticeably, there is a need for more validated tools to measure obesity-related parenting practices.

The critical gap that this study aims to address is the use of adolescents as proxy reporters on their parents’ obesity-related parenting practices. Research on parenting practices presents a host of challenges, and proxy reports (especially from the direct recipients of those parenting practices) offers a potential way to increase response rates and reduce costs and time associated with the collection of these data [23, 31]. In assessing the validity of adolescent reports of obesity-related parenting, our research team expects to provide a new avenue for research on obesity influences in the family context by introducing key validated measurements that allow for more predictive power of adolescents’ obesity-related outcomes.

This study has two central aims. First, to assess the validity and reliability of a measurement tool of obesity-related parenting practices, both through parent and adolescent reports. Our second aim is to test the associations between the obesity-related parenting practices and energy balance behaviors (i.e., diet and physical activity), and compare the strengths of these associations between parent and adolescent reports. We hope to reach conclusions about the validity and usefulness of adolescent proxy reports of obesity-related parenting practices that will inform future research on obesity prevention.

## Methods

### Study Sample

This study utilizes data collected in the National Cancer Institute’s Family Life, Activity, Sun, Health, and Eating (FLASHE) study. This cross-sectional study administered a web-based survey to investigate psychosocial and environmental correlates of eating and physical activity behaviors among parents and adolescents separately. Adolescents were eligible for participation if they were between ages 12 and 17 and lived with the parent more than 50% of the time, and parents were eligible for participation if they were at least 18 years of age and lived with the adolescent more than 50% of the time, per the inclusion criteria of the FLASHE study. The sample included 1,849 parent-adolescent dyads who were recruited from across the United States via print ads, internet banner ads, random digit dialing omnibus surveys, and panelist referrals and screened for eligibility [32]. The sample was collected to reflect the US population distribution based on sex, census division, household income, household size, among others. The FLASHE study utilized two surveys, one collecting data on diet and one collecting data on physical activity, along with a demographic survey attached to whichever survey was completed first. Surveys were conducted between April and October of 2014 [33].

### Measures

#### Parenting practices

The current study analyzes variables related to parenting practices in four behavioral domains: 1) fruit and vegetable intake, 2) junk food and sugar beverage intake, 3) physical activity, and 4) screen time. Parenting for fruit and vegetable intake was assessed through seven (7) items adapted from the Child Feeding Questionnaire, the Comprehensive Feeding Questionnaire, the Parental Feeding Style Questionnaire, Legitimacy of Parental Authority, with one new survey item created for this study [34–37]. Parenting for junk food and sugary drink intake was assessed through seven (7) items adapted from the same four sources as the fruit and vegetable survey items. Physical activity parenting was assessed through six (6) items adapted from the Parenting Eating and activity Scale, the Activity Support Scale, the Comprehensive Feeding Practices Questionnaire, and the Legitimacy of Parental Authority, with one new survey item created for this study [35, 36, 38, 39]. Screen time parenting was assessed through seven (7) items adapted from the same four sources as the physical activity survey items. Item response options across five-point Likert scales ranged from strongly disagree to strongly agree.

### Energy balance behaviors

Energy balance behaviors (i.e., fruit, vegetable intake, sugar) were taken from self-reports on adolescents’ and parents’ behaviors in each of these four domains representing the behaviors the parenting practices aimed to promote or prevent, i.e., daily servings of fruits and vegetables or weekly screen time. The outcome variable for fruit and vegetable intake was generated from responses on two questions asking how many times in the past 7 days the respondent consumed fruits and vegetables, respectively. Their responses to these two questions were divided by seven and added together to yield a total average daily intake of fruits and vegetables. The outcome variable for junk food and sugary beverage intake was generated similarly from weekly responses of intake of junk food and sugary beverages. The outcome variable for physical activity was generated by taking the sum of self-reported weekly minutes spent doing moderate physical activity and twice the self-reported weekly minutes spent doing vigorous physical activity using the International Physical Activity Questionnaire calculations. The Centers for Disease Control and Prevention recommends 150 weekly minutes of moderate-to vigorous activity for adults and 60 min per day for adolescents [40]. The outcome variable for screen time was generated by taking the sum of the self-reported daily time spent on a phone, TV, computer, or video game.

#### Demographics

Both adolescents and parents completed a demographic survey. For the adolescents, demographic variables include sex, age, race, and ethnicity, and for the parents, demographic variables include sex, age, education level, race, and ethnicity.

### Statistical analysis

Statistical analysis was conducted on STATA, version 16, between August 2020 and March 2021. Statistical significance was set at *p* < 0.05. Initially, univariate descriptive statistics were obtained for the demographic variables and the variables of all four parenting domains. Subsequently, bivariate statistics (i.e., Pearson’s Correlation, OLS regression) were used to assess the relationship between parent- and adolescent-reported obesity parenting at the item and at the domain level. Next, internal consistency for each parenting domain, at the individual level (parent or adolescent separately), was assessed using Cronbach’s alpha [41]. Items were reverse coded where appropriate so that, for each item, a higher number indicates parenting that promotes the behavior, and a lower number indicates parenting that restricts or inhibits said behavior. Next, factorial validity was tested for each of the four obesity parenting domains, as well as measurement invariance across adolescent and parent reports using confirmatory factor analysis. For the confirmatory factor analysis models and invariance models, goodness of fit was assessed by collecting the root mean square error of approximation, standardized root mean square residual (both absolute measures of fit), and the Comparative Fit Index (incremental measure of fit). Model fit was acceptable if it met two of the following three criteria: A root mean square error of approximation less than or equal to 0.08, a standardized root mean square residual of less than 0.10, and a comparative fit index of greater than 0.90 [42]. Lastly, predictive validity was assessed using Actor-Partner Interdependence Modeling (APIM), where adolescent and parent responses on obesity-related parenting were associated to behavioral outcomes. The APIMs were adjusted for parent sex and education level.

## Results

The study sample consisted of 1,859 total adolescent-parent dyads. Due to some missingness, some demographic categories may not sum to 100% of the study sample. Of the parents, 71.6% were mothers and 25.3% were fathers. Most parents were between the ages of 35 and 44 (43.6%), as well as between the ages of 45 and 59 (42.3%). Nearly half of the parents in the study had a four-year college degree or higher (46.5%). Over a third of the parent sample identified as Non-Hispanic (NH) White, while 17.7% were NH Black or African American and 7.3% were Hispanic/Latino. In the adolescent sample, 45.1% were males and 45.5% were females ranging in age from 12 to 17 years old. Nearly a third of the adolescent sample were NH White (63.7%), while 17.0% were NH Black or African American and 10.0% were Hispanic/Latino. The remaining demographic characteristics are summarized in Table 1.

**Table 1:** Descriptive statistics of the study sample (*n* = 1,859)

Characteristics	Parents	Adolescents
**Sex, *n* (%)**		
Female	1,325 (71.6)	843 (45.5)
Male	468 (25.3)	835 (45.1)
**Age, *n* (%)**		
12	-	224 (13.3)
13	-	336 (20.0)
14	-	280 (16.7)
15	-	305 (18.1)
16	-	331 (19.7)
17	-	206 (12.3)
18–34	202 (11.3)	-
35–44	781 (43.6)	-
45–59	758 (42.3)	-
60 +	52 (2.9)	-
**Highest education level, *n* (%)**		
Less than high school	22 (1.2)	-
High school degree or Equivalent	301 (16.8)	-
Some college	634 (35.5)	-
Four-year degree or higher	830 (46.5)	-
**Race and ethnicity, *n* (%)**		
Hispanic	130 (7.3)	168 (10.0)
Non-Hispanic Black or African American	314 (17.7)	283 (17.0)
Non-Hispanic White	1,229 (69.1)	1,061 (63.7)
Non-Hispanic other race or ethnicity	105 (5.9)	154 (9.2)
**Energy balance behaviors, Mean (*SD*)**		
Fruit and vegetable intake (average servings consumed daily)	1.8 (1.3)	1.5 (1.2)
Physical activity (average weekly minutes)	269.1 (29.3)	114.6 (21.9)
Junk food and sugary drinks intake (average servings consumed daily)	1.6 **(**3.7**)**	2.1 (4.4)
Screen time (average daily hours)	8.1 (5.0)	5.1 (4.0)

Due to missing data, some of these categories will not add up to the total number of survey respondents

At the item level, adolescents’ reports of obesity parenting were correlated with their parents’ reports across all four domains (*r* = 0.31–0.58). Only three items within the physical activity and screen time parenting were not retained with coefficients below 0.30 (*r* = 0.27–0.29; β = 0.00–0.24). At the domain level, adolescents’ reports of obesity parenting were correlated with their parents’ reports across all four domains (*r* = 0.57–0.62). In regression, adolescents’ report of obesity parenting was associated with their parents’ report across all four domains (β = 0.59–0.70; *p* < 0.001; *R*^2^ = 0.32–0.37) (Table 2).

**Table 2:** OLS regression models between adolescent- and parent-reported obesity-related parenting practices (*n* = 1,591)

Parenting Domain	Alpha (SE)	Beta (SE)	*T* statistic *p* value	R^2^
Fruit and vegetable parenting	0.99 (0.09)	0.70 (0.02)	0.01	0.34
Junk food parenting	0.64 (0.08)	0.64 (0.02)	0.01	0.32
Physical activity parenting	0.93 (0.07)	0.66 (0.02)	0.01	0.37
Screen time parenting	1.03 (0.07)	0.59 (0.02)	0.01	0.32

All OLS regression models were unadjusted. Adolescent-reported parenting practices served as the independent variable in all models, whereas parent-reported obesity-related parenting practices served as the dependent variable. Due to missingness, the sample size at baseline was reduced in these models using listwise deletion approach

Results from CFA models (Table 3) show that obesity parenting indicators significantly loaded onto all four respective domains in adolescents and parents (β = 0.45–0.85; *p* < 0.001; CFI = 0.93–0.99; SRMR = 0.01–0.05), which meets goodness of fit criteria. With regards to measurement invariance, CFA results only supported a single factor screen time parenting model that met metric invariance between parents and adolescents (ΔCFI = 0.05; ΔRMSEA = 0.01; ΔSRMR = 0.03).

**Table 3:** CFA results for the final factor structure for each obesity-related parenting domain (*n* = 1,765; *p* < 0.05)

Survey Item (shown as on parent survey)	Parents	Adolescents
**Fruit and Vegetable Parenting**		
1. I buy fruits and vegetables for my teenager	0.54	0.52
2. I try to eat fruits and vegetables when my teenager is around	0.62	0.63
3. I encourage my teenager to try different kinds of fruits and vegetables	0.67	0.62
4. My teenager and I decide how many fruits and vegetables he/she has to eat	0.64	0.72
5. I have to make sure that my teenager eats enough fruits and vegetables	0.51	0.67
6. I make my teenager eat fruits and vegetables	0.57	0.62
7. It’s okay for me to make rules about how many fruits and vegetables my teenager can have	0.52	0.62
RMSEA	0.12	0.14
CFI	0.94	0.94
SRMR	0.04	0.06
**Junk Food Parenting**		
1. If my teenager has a bad day, I let him/her have junk food/sugary drinks to feel better	-	-
2. I don’t buy a lot of junk food or sugary drinks for my teenager	-	-
3. I try to avoid junk food or sugary drinks when my teenager is around	0.32	0.45
4. My teenager and I decide together how much junk food or sugary drinks he/she can have	0.55	0.81
5. I have to make sure that my teenager doesn’t eat too much junk food or drink too many sugary drinks	0.80	0.64
6. I decide how much junk food or sugary drinks my teenager can have	0.82	0.73
7. It’s okay for me to make rules about how much junk food or sugary drinks my teenager can have	0.54	0.59
RMSEA	0.03	0.04
CFI	0.99	0.99
SRMR	0.01	0.01
**Physical Activity Parenting**		
1. I have to make sure my teenager gets enough physical activity	0.61	0.66
2. I take my teenager places where he/she can by physically active	0.54	0.61
3. My teenager and I decide together how much physical activity he/she has to do	0.69	0.85
4. I make my teenager exercise or go out and play	0.79	0.79
5. I try to be physically active when my teenager is around	0.67	0.65
6. It’s okay for me to make rules about how much time my teenager spends being physically active/playing	0.57	0.54
RMSEA	0.12	0.15
CFI	0.95	0.97
SRMR	0.04	0.04
**Screen Time Parenting**		
1. If my teenager has a bad day, I let him/her have screen time to feel better	-	-
2. My teenager and I decide together how much screen time he/she can have	0.63	0.78
3. I take my teenager places where he/she can play video games, watch movies, etc	-	-
4. I decide how much screen time my teenager can have	0.88	0.80
5. I have to make sure my teenager does not have too much screen time	0.79	0.70
6. I try to limit my own screen time when my teenager is around	0.58	0.67
7. It’s okay for me to make rules about how much screen time my teenager can have	0.63	0.64
RMSEA	0.15	0.00
CFI	0.99	1.00
SRMR	0.02	0.00

Due to missingness, the sample size at baseline was reduced in these models using listwise deletion approach

Results from the APIM analyses are summarized in Table 4. The standardized parameter estimates are reported to compare models across parenting domains. Regarding actor effects, both parent- and adolescent-reported parenting practices for fruit and vegetable consumption were significantly associated with their own fruit and vegetable intake (β = 0.21–0.32; *p* < 0.05). In addition, adolescent-reported physical activity parenting practices were significantly associated with adolescent physical activity (β = 0.23; *p* < 0.05). Regarding partner effects, only parent-reported parenting practices for fruit and vegetable consumption were significantly associated with adolescent intake of fruit and vegetables (β = 0.15, *p* < 0.05) and adolescent-reported physical activity parenting practices were significantly associated with parental physical activity (β = 0.16, *p* < 0.05). Neither adolescent nor parent reported parenting practices were significantly associated with screen time or junk food intake outcomes.

**Table 4:** APIM models assessing the interdependent associations between obesity-related parenting practices and energy balance behaviors among parent-adolescent dyads. (*n* = 1,583)

	**Fruit and Vegetable**		**Junk Food**		**Physical Activity**		**Screen Time**	
Dyad pathway	Actor effect	Partner effect	Actor effect	Partner effect	Actor effect	Partner effect	Actor effect	Partner effect
Parent-adolescent	0.32	0.15	–	–	–	–	–	–
Adolescent-parent	0.21	–	–	–	0.23	0.16	–	–

All standardized parameter estimates presented in the table are significant at *p* < 0.05

Table 5 describes internal consistency for each factor (Cronbach’s alpha) as well as summary statistics for each item in the final factor structure across parenting domains. There was good internal consistency across parenting domains among both adolescents (*a* = 0.79–0.85) and parents (*a* = 0.74–0.83).

**Table 5:** Descriptive and internal consistency statistics for obesity-related parenting domains

Survey Item (shown as on parent survey)	Mean (SD)	
	**Parents**	**Adolescents**
**Fruit and Vegetable Parenting**		
1. I buy fruits and vegetables for my teenager	4.47 (0.76)	4.47 (0.83)
2. I try to eat fruits and vegetables when my teenager is around	4.16 (0.94)	4.09 (1.05)
3. I encourage my teenager to try different kinds of fruits and vegetables	4.44 (0.74)	4.35 (0.89)
4. My teenager and I decide how many fruits and vegetables he/she has to eat	3.16 (1.22)	3.09 (1.31)
5. I have to make sure that my teenager eats enough fruits and vegetables	3.92 (1.17)	3.44 (1.32)
6. I make my teenager eat fruits and vegetables	3.36 (1.27)	3.37 (1.36)
7. It’s okay for me to make rules about how many fruits and vegetables my teenager can have	3.85 (1.06)	3.51 (1.20)
Cronbach’s alpha	*a* = 0.78	*a* = 0.83
**Junk Food Parenting**		
1. If my teenager has a bad day, I let him/her have junk food/sugary drinks to feel better	3.64 (1.14)	3.58 (1.18)
2. I don’t buy a lot of junk food or sugary drinks for my teenager	3.55 (1.19)	3.37 (1.24)
3. I try to avoid junk food or sugary drinks when my teenager is around	3.20 (1.23)	3.12 (1.26)
4. My teenager and I decide together how much junk food or sugary drinks he/she can have	3.17 (1.18)	3.04 (1.25)
5. I have to make sure that my teenager doesn’t eat too much junk food or drink too many sugary drinks	3.60 (1.24)	3.22 (1.32)
6. I decide how much junk food or sugary drinks my teenager can have	3.44 (1.17)	3.20 (1.26)
7. It’s okay for me to make rules about how much junk food or sugary drinks my teenager can have	4.09 (.948)	3.54 (1.16)
Cronbach’s alpha	*a* = 0.74	*a* = 0.79
**Physical Activity Parenting**		
1. I have to make sure my teenager gets enough physical activity	3.30 (1.34)	2.98 (1.32)
2. I take my teenager places where he/she can by physically active	3.67 (1.15)	3.65 (1.19)
3. My teenager and I decide together how much physical activity he/she has to do	2.82 (1.22)	2.75 (1.27)
4. I make my teenager exercise or go out and play	3.07 (1.31)	2.98 (1.33)
5. I try to be physically active when my teenager is around	3.45 (1.11)	3.18 (1.24)
6. It’s okay for me to make rules about how much time my teenager spends being physically active/playing	3.72 (1.02)	3.30 (1.34)
Cronbach’s alpha	*a* = 0.80	*a* = 0.79
**Screen Time Parenting**		
1. If my teenager has a bad day, I let him/her have screen time to feel better	3.20 (1.21)	3.25 (1.24)
2. My teenager and I decide together how much screen time he/she can have	2.95 (1.20)	2.78 (1.29)
3. I take my teenager places where he/she can play video games, watch movies, etc	3.49 (1.29)	3.34 (1.30)
4. I decide how much screen time my teenager can have	3.24 (1.26)	2.94 (1.36)
5. I have to make sure my teenager does not have too much screen time	3.43 (1.27)	3.02 (1.36)
6. I try to limit my own screen time when my teenager is around	3.00 (1.25)	2.59 (1.29)
7. It’s okay for me to make rules about how much screen time my teenager can have	4.06 (0.97)	3.25 (1.23)
Cronbach’s alpha	*a* = 0.82	*a* = 0.85

Each item was answered on a Likert scale from 1 to 5, with a 1 indicating strongly disagree and a 5 indicating strongly agree

## Discussion

Given the public health risks associated with obesity and the strong demonstrated influence that parents can have on their children’s energy balance behaviors – one of the most proximal determinants of obesity – understanding and improving obesity-related parenting practices is warranted [13–19]. Advancing current and future obesity-prevention efforts necessitates the reduction of gaps and barriers to effective measurement of such parenting practices.

Obesity-related parenting practices must be measured more consistently and validly [30, 43, 44], but researchers must also address the challenges of gathering parenting data such as cost, time intensiveness, and low response rates, particularly if populations are hardly reached [23, 31].

This study aimed to assess the role of proxy reports from the recipients of obesity-related parenting practices (i.e., adolescent children) as prospective obesity parenting measurement targets. We found that adolescent responses were significantly correlated with parent responses on their obesity-related parenting practices across parenting domains, and each construct showed strong internal consistency. Also, we found statistically significant invariance in the screen time parenting measure across groups. Lastly, actor-partner effects showed that both parent- and adolescent-reported parenting practices for fruit and vegetable consumption were associated with parent- and adolescent-reported fruit and vegetable intake (3 out of 4 pathways), and only adolescent-reported physical activity parenting practices were associated with parental physical activity and their own physical activity. It is also worth pointing out that parents reported more than twice as many minutes spent in physical activity than their adolescents did, which is a substantial difference in activity levels and should be investigated further in subsequent studies.

Establishing the validity of these four obesity-related parenting domains addresses a significant gap in the literature on obesity-related parenting practices. Systematic reviews conducted by Vaughn et al. and Davison et al. on the measurement of diet-related and physical activity-related parenting practices, respectively, demonstrated need for obesity-related parenting practices measurement tools with established validity [30, 44]. An additional review study of physical activity parenting measurement found widespread use of non-validated tools [43]. With the validation approach taken in our study, we hope future research adopts well validated tools to measure obesity-related parenting practices.

Adolescents’ reports on their parents’ obesity-related parenting practices were also evaluated with regards to their similarity to their parents’ reports on the same obesity-related parenting practices. Our findings show that they are significantly associated. This is indicative of the relative congruity between adolescent proxy-reports and parent self-reports of their obesity-related parenting practices. While the literature is limited, our validation results support prior findings that adolescent proxy reports may be a valid source of information on obesity-related parenting practices. Previous research has found child proxy reports of parents’ behaviors are less accurate than self-report, but also showed that accuracy increased as children were older [31, 45, 46]. Additionally, much of this work was investigating reports on socioeconomic status, not parenting practices, and there is evidence to suggest that child proxy reports are more accurate in areas that are more salient to them [31]. Considering that obesity-related parenting practices are reasonably salient to adolescents, our findings support past research and suggest that adolescents are reliable sources of information on obesity-related parenting practices.

By modeling actor-partner effects, we found that both adolescent and parent reports on fruit and vegetable parenting practices were predictive of adolescent fruit and vegetable intake, but adolescent reports yielded a higher slope coefficient at a higher significance level than parent reports. With physical activity, only adolescent reports of parenting practices predicted adolescent outcomes. Only one small study has assessed parent-practices in parent–child dyads, but it found that child reports of obesity-related parenting practices were associated with child diet and physical activity, but parent reports were not [47]. There are two major hypothesized reasons why adolescent proxy reports on parenting are more predictive of behavioral outcomes: parent reports may be subject to social desirability bias, and the effects parenting practices have on adolescent behaviors likely be contingent in adolescent perceptions of them [48]. As such, our findings suggest that parent- and adolescent-reported parenting practices correlated very well, thus parent-reporter bias is unlikely to have had an impact in the study outcomes.

This finding that adolescent proxy reports could potentially be more predictive of adolescent energy balance behaviors has substantial implications for future research on obesity-related parenting practices. The primary justification for studying obesity-related parenting practices is the strong influence they may have on children’s energy balance behaviors. Our findings suggest that adolescent-reported parenting practices are more predictive of highly pertinent behavioral outcomes than those that are parent-reported, and as such, might be a relatively more accurate source of parental influence, rather than simply a valid alternative proxy. Future work should be conducted to further clarify and confirm this disparity of predictivity between adolescent and parent reports and the reasons for this disparity, as well why neither adolescent nor parent reported screen time and junk food parenting practices were predictive of relevant behavioral outcomes.

There have long been issues with measurement of parenting practices, including but not limited to cost, time, response rate, and bias [23]. Adolescent proxy reports could potentially address some of these barriers [31]. The ability to assess adolescent behavior in combination with adolescent proxy reports of parenting practices would negate the need for parental report. Furthermore, the existence of schools and other community settings as centralized places to partner with, recruit, and follow up with adolescents may make them a study population that is feasibly approachable. In these ways and others, using adolescent proxy reports could offer an avenue that makes research on parenting practices more feasible, and could open doors to research on otherwise hard to reach populations. These findings are also promising for future family-based obesity prevention strategies, as this indicates that adolescents could be valuable stakeholders in obesity prevention research and interventions.

There are several strengths to this study. First, data was taken from a large sample that was selected to be demographically representative of the U.S. population. This allows for generalizability of the findings in this study at the population level. Furthermore, having dyadic data from parents and their adolescent children allowed for direct comparisons, ensuring that the parent-adolescent reports were on the same parenting practices. Furthermore, including four obesity-related parenting domains allowed our team to get a more complete picture of this concept and how adolescents report on them, rather than focusing in on behavioral domains independently. Another important strength of this study is the variety of validation steps that were taken to ensure the parenting practices measures included in our study were rigorously validated; this is contrasted with past studies that have often used unvalidated tools [43].

There are a few limitations to this study that are important to mention. The main outcome measures rely on self-report data, which can be vulnerable to social desirability bias, as respondents may feel they should answer to reflect what they should be doing rather than what they actually do. Past research has shown that on self-reported questionnaires, social desirability bias can lead to underreporting of eating and overreporting of physical activity [49, 50]. In the context of this study, there may be different factors affecting parents’ social desirability bias compared to adolescents, which ultimately have a varying degree of risk. This might partly explain the disparity seen in activity levels between parents and adolescents in the study sample. Furthermore, reports on energy balance behaviors in our study can also be subject to recall bias; past research has shown that asking for self-reports of physical activity in the past can be flawed when compared to data collection of more recent physical activity, such as in the form of a daily diary [51]. An additional limitation is that our study sample is U.S. based, and although it may a demographically representative of the U.S. population, these findings may not be as generalizable to other populations. Lastly, our study did not assess sex concordance between parents and adolescents which some evidence suggests it is relevant to the influence of parenting practices [52, 53], which future studies should account for this contribution.

## Conclusion

The goal of this study was to assess the factorial and predictive validity of adolescents’ proxy-report of parents’ obesity-related parenting practices across four behavioral domains. We found that adolescent- and parent-reported obesity-related parenting practices were significantly associated, while adolescent-reported parenting practices were more informative of fruit and vegetable intake and physical activity than parent-reported parenting practices. Future work should consider adolescents as key stakeholders of family-based obesity research and prevention efforts.

## Data Availability

The datasets generated and/or analyzed during the current are available and retrieved from: https://cancercontrol.cancer.gov/brp/hbrb/flashe-study/flashe-files.

## Abbreviations

NH: Non-Hispanic
FLASHE: Family Life, Activity, Sun, Health, and Eating
